# Influence of the Grain Orientation of Wood upon Its Sound Absorption Properties

**DOI:** 10.3390/ma16175998

**Published:** 2023-08-31

**Authors:** Maria Violeta Guiman, Mariana Domnica Stanciu, Ioan Călin Roșca, Sergiu Valeriu Georgescu, Silviu Marian Năstac, Mihaela Câmpean

**Affiliations:** 1 Department of Mechanical Engineering, Transilvania University of Brașov, 500036 Brașov, Romania; icrosca@unitbv.ro; 2 Faculty of Furniture Design and Wood Engineering, Transilvania University of Brasov, 500036 Brașov, Romania; sergiu.georgescu@unitbv.ro (S.V.G.); campean@unitbv.ro (M.C.); 3 Faculty of Engineering and Agronomy, Braila, “Dunarea de Jos” University of Galati, 800008 Galati, Romania

**Keywords:** sound absorption coefficient, sound reflection coefficient, impedance ratio, porosity, wood

## Abstract

The purpose of the study was to analyze the influence of the quality class and the orthotropy of wood upon the sound absorption coefficient, the reflection and the impedance ratio of two species widely used for stringed musical instruments, namely spruce (*Picea abies* L. *Karst*) and maple (*Acer pseudoplatanus* L.). An impedance tube for the frequency range 100–6400 Hz was used in these experimental determinations. Knowing the influence of porosity and tortuosity on the acoustic absorption, these properties were also determined, as well as the sound reduction coefficient and the maximum values of the acoustic absorption coefficients in relation to frequency. The main results highlighted the differences between the anatomical quality class of the wood within each species, but also concerning the sound direction relative to the three main sections of wood, as an orthotropic material. The article highlights the acoustic performance parameters related to the frequency of the wooden material and its relationship to density, porosity and quality class. The results represent useful information for musical instruments manufacturers and more.

## 1. Introduction

Wood, by its biological nature, has a porous structure; it represents a semi-phenomenological model with a motionless skeleton having arbitrary pore shapes, according to the types of sound-absorbing material models established by [1,2,3,4]. Moreover, the structure of wood differs in the three main directions: longitudinal, tangential and radial. Considering the wood structure, the three axes of elastic symmetry are: longitudinal L, radial R and tangential T, and the three planes of elastic anisotropy are LR, RT and TR [1,5]. The principle of sound energy absorption from porous materials, described by [6], is based on the following mechanical, thermal and acoustic phenomena: (a) in the first stage, from the interaction between the vibrating air molecules and the pore walls of the porous materials, sound energy conversion into heat occurs and its dissipation; (b) in the next stage, through the penetration of longitudinal sound waves into the material, the air inside the pores is compressed and decompressed, resulting in energy consumption; (c) and in the third stage, sound energy is transformed into mechanical and thermal energy through the vibration of the pore walls [7]. Depending on the number of pores (cavities, channels, interstices), their sizes and the connections between the pores, the interface between the outer surface of the materials and the inner pores, several models have been developed to predict acoustic absorption by porous materials [8,9,10]. Depending on the morphology of the material and the number of parameters considered in the calculation expressions, these analytical models have been developed starting from empirical models of materials with straight cylindrical pores, to materials with inclined pores with a circular section, to materials with pores with a section non-uniform, up to porous materials with a non-uniform cross section and partial constrictions [10,11,12,13,14].

Wood is an antagonistic material, some species being used for their acoustic properties in musical instruments and others for their sound-absorbing characteristics [15,16,17,18].

Studies by [19] on wood samples’ cross sections exposed to the flow of acoustic waves highlighted that the sound absorption coefficient of hardwood is different due to its different crystallinity, as well as different morphological characteristics, depending on the micropores structure and gas permeability. Thus, at 2 kHz, the highest values of the acoustic absorption coefficient have been found for plane wood and silver poplar wood (0.432–0.464); and at 4 kHz, for hackberry, light balsa, platanus and maple wood (0.646; 0.557; 0.450; 0.314). Jang et al. [20] highlighted the connection between the sound absorption coefficients of wood and its permeability, noting that very permeable wood specimens also show high values of the acoustic absorption coefficient because the increased air flow contributes to the attenuation of the sound waves.

The studies carried out by Smardzewski et al. [10] to determine the acoustic absorbing characteristics of 10 wood species (from the temperate zone as well as exotic species), measured both along the longitudinal–tangential direction and along the longitudinal–radial direction, highlighted that the highest sound absorption coefficients for the frequency of 2 kHz were recorded for oak (LT), ash (LT), sapele (LR), pine (LR) and ash (LR), varying between 0.18 and 0.20. The authors of the study analyzed the influence of the different parameters that influence the acoustic absorption. They also carried out a numerical study based on the existing calculation models in the literature. Thus, the density, porosity and tortuosity of the wood species led to a significant increase in the sound absorption coefficient, especially at frequencies of 2 and 4 kHz. Certain researchers have studied thin cork panels and showed that samples with a thickness of 1.5 mm achieve a measured sound absorption coefficient value of 0.8 for the frequency of 1 kHz [21].

It is certain that most of the studies on wood samples have mainly analyzed the values of the acoustic absorption coefficient at high frequencies (2–6 kHz), the sound absorption characteristics of which vary significantly depending on the frequency, material thickness, porosity and angle of incidence of the sound [22,23,24]. In the construction of stringed musical instruments, the sound box must have a high damping, and therefore a large resonant field, equally amplifying a wide spectrum of frequencies. In this sense, the wood used in the construction of the resonating body plays a double role: it requires a high speed of propagation of vibrations/sound- but also a sound damping capacity so as to allow the reception and amplification of the following frequencies during musical interpretation [5,6,24]. Wood is a material that presents stochastic, complex and sinuous microstructures, as [25,26] characterizes open cell materials such as foams. Wood is a material known for its acoustic properties, being used as both a sound insulating material and as a material with resonance properties in the construction of musical instruments. Although most of the studies on the wood used for musical instruments theoretically and experimentally approach the determination of the acoustic properties from the perspective of the sound propagation speed, resonance frequencies, damping and quality factor, the current study in this paper approaches an inverse problem of these research works. Knowing the acoustic characteristics, including the sound absorption coefficient of the soundboard, is important in predicting the amount of sound absorbed by the violin’s resonance body. The work in [6,7,8,9,10,27] highlighted the hypothesis that there is an inversely proportional relationship between the value of the specific modulus and the damping in the longitudinal direction in the resonance wood. However, in the radial and tangential directions, this hypothesis has not yet been demonstrated. Since these directions also influence the dynamics of stringed musical instruments, the usual selection criteria are controversial according to [6], requiring more in-depth study on the acoustic and elastic properties of the soundboard in the other directions (radial and tangential).

Thus, the objectives of the paper are to answer the following questions: what are the frequencies at which the acoustic absorption is at a maximum, how it is influenced by the main sections of the wood and to what extent does the quality of the anatomical structure of the wood influence these acoustic properties? From the studies thus far, this is the only one that addresses, for the first time, the absorption and acoustic reflection properties of the wood used for musical instruments.

Starting from these principles related to the acoustic quality of a violin (for example), the study aims to analyze the relationship between the orthotropy of wood and the sound absorption coefficient. In this sense, two wood species used for musical instruments were analyzed—spruce (*Picea abies* L. *Karst*) and maple (*Acer pseudoplatanus* L.)—each species being classified into two quality classes (based on some specific anatomical features) and cut according to the three planes of elastic symmetry of the wood, according to previous studies [3,4].

The novelty of the study consists in identifying the frequencies at which the acoustic absorption coefficient has extreme values, and correlating the data with wood porosity and wood tortuosity for each of the three main sections of the wood samples. The practical importance of the study consists not only in gathering the information regarding the soundboard, information that has not been reported thus far in the specialized literature, but also in understanding the phenomena that appear at the wood–acoustic wave interface in the structure of musical instruments with a resonator body.

## 2. Materials and Methods

### 2.1. Sample Preparation

Two wood species of major importance for stringed musical instruments manufacturing were selected for this research: spruce and maple. The material was purchased from the warehouse of a Romanian violin factory. It originated from trees harvested from the Gurghiu area, Eastern Carpathian mountains. The samples were sorted according to their anatomical features in two quality classes: class A, representing wood of superior anatomical quality, used for maestro and professional musical instruments, and class D, representing lower quality wood, used for musical instruments for beginners. From an anatomical point of view, the class A spruce wood samples are characterized by an annual rings width lower than 1.5 mm, with a proportion of latewood below 30%, compared to class D spruce wood, where the width of the annual rings is less than 3 mm, and the proportion of latewood is below 45% [3]. Class A maple wood has an annual rings width below 1.3 mm and a curly grain, with a pitch length of undulation below 3 mm, compared to class D maple wood, with a width of the annual rings below 2.5 mm and a straight fiber [4].

In the first stage, the samples were obtained as cubes with a side size of 40 mm. These samples were analyzed in terms of the variability of the anatomical characteristics (width of the annual rings, the proportion of latewood/earlywood, the undulation pitch of the curly grain for the maple wood), and regarding their acoustic and elastic properties by the US method that has been presented extensively in previous publications [3,4].

**Figure 1:**
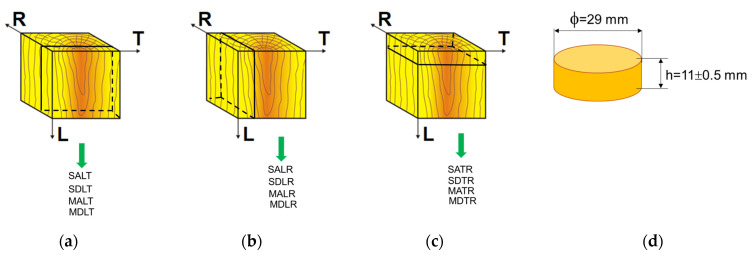
The method of extracting cylindrical samples for the impedance tube test from cubes: (**a**) the sample with a longitudinal–tangential section; (**b**) the sample with a longitudinal–radial section; (**c**) the sample with a cross section; (**d**) the geometry of the sample for the impedance tube.

In the second stage, the cubes were cut into prisms of equal thickness (11 ± 0.5 mm), respecting the main sections of the wood (Figure 1). Finally, to obtain the cylindrical shape, the prisms were laser cut, the following codifications of the samples being used: SALR—spruce sample, class A, longitudinal–radial section; SALT—spruce sample, class A, longitudinal–tangential section; SATR—spruce sample, class A, cross section; SDLR—spruce sample, class D, longitudinal–radial section; SDLT—spruce sample, class D, longitudinal–tangential section; SDTR—spruce sample, class D, cross section; MALR—maple sample, class A, longitudinal–radial section; MALT—maple sample, class A, longitudinal–tangential section; MATR—maple sample, class A, cross section; MDLR—maple sample, class D, longitudinal–radial section; MDLT—maple sample, class D, longitudinal–tangential section; MDTR—maple sample, class D, cross section. Thus, ten specimens for each category were obtained.

### 2.2. Methods

#### 2.2.1. Density, Porosity and Tortuosity Determination

In the first stage, the cylindrical samples prepared for the impedance tube were weighed with an accuracy of ±0.2% and measured with the electronic device, determining their diameter and thickness, and based on the formula, the apparent density was calculated at a humidity of 6–8%. Afterwards, the samples were placed in the oven and gradually dried until they reached a constant mass. After cooling in the desiccator, the samples were notched and measured, and based on the ratio between the mass and volume, the value of the density of the samples in the absolutely dry condition was determined, according to ISO 13061-2/2014 [28].

Porosity can be determined by weight measurements, the buoyancy method, or mercury intrusion porosimetry [29,30], but in this research we calculated the porosity and tortuosity based on the formulas extracted from references. The porosity (denoted $D_{w}$) is calculated by Equation (1), as a function of the oven-dry density of the samples ($\rho_{0})$ and the cell wall density ($\rho_{cw})$, which varies between 1460 kg/m^3^ and 1530 kg/m^3^. For the calculation, the value of the cell wall density of 1500 kg/m^3^ was adopted, according to the references [10,25,26,27]:

(1) $$D_{w}=1-\frac{\rho_{0}}{\rho_{cw}},$$

The samples were oven-dried at 103 °C, and then the oven-dry density was determined by the method of weighing and measuring the dimensions of the samples. According to [10,25,26,27,28,29,30], tortuosity refers to the degree of deviation of the fibers from a straight direction, and it applies to porous materials.

The tortuosity denoted $k_{s}$ was determined based on Equation (2) established by [10,27]:

(2) $$k_{s}=\frac{1}{\sqrt{D_{w}}},$$

#### 2.2.2. Sound Absorption Coefficient

To determine the sound absorption coefficient, the acoustic impedance and the admittance, reflection and transmission loss coefficients, the impedance tube method (also known as the Kundt tube method) was used. The testing procedure is described in [31,32,33,34,35,36]. The theoretical principle of this method is based on the evaluation of the field of stationary plane waves propagated in a tube which split into incident waves and reflected waves when meeting the material sample in the tube [37,38,39,40]. This is how the values of the minimum and maximum acoustic pressure levels are measured.

The analyses in impedance tube were performed related to the standard procedure for measurements based on the two-microphone transfer function method (TFM) according to the ISO 10534-2 and ASTM E1050-12 international standards [36,37,38]. Hereby, the transfer function method (TFM) was used in order to evaluate the acoustic impedance and sound reflection coefficient at a normal impedance (the sound absorption coefficient was computed directly from the reflection parameter); this representation in terms of real and imaginary parts mainly reveals the two main aspects of surface behavior at sound striking such as resistance (real component) and reactance (imaginary component), respectively.

**Figure 2:**
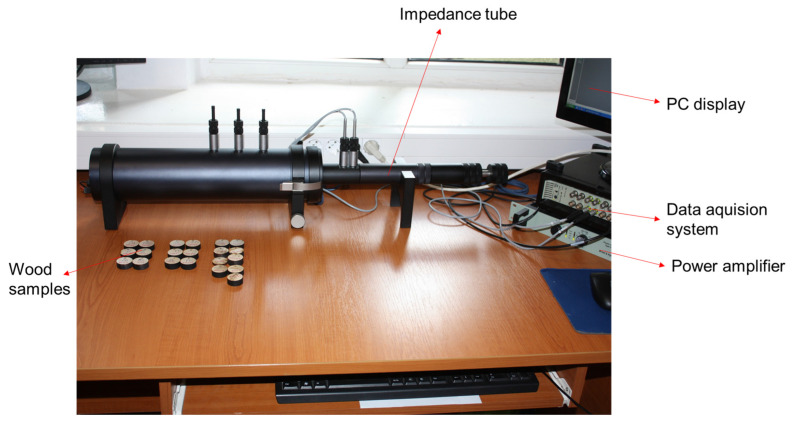
Experimental setup of acoustic measurement.

The measurements were made with an acoustic system consisting of an impedance small tube Type 4206 Brüel & Kjær (Nærum, Denmark) (sample diameter of 29 mm), with a frequency range of 50 Hz–6.4 kHz. The schematic experimental setup of acoustic measurement is shown in Figure 2. Before the measurements, the microphones and the whole device are calibrated, and after each step of determining the acoustic characteristics, they are recalibrated. Data acquisition and visualization were performed with the Pulse Brüel & Kjær software (Nærum, Denmark), a series of parameters required by the data processing program being introduced in the pre-processing stage [41,42,43]. Each sample was fixed to the inner wall of the tube.

According to [33,34,35], after the sound absorption coefficient (noted α) was determined, the noise reduction coefficient, NRC, was calculated as the average of the sound absorption at specific mid-range frequencies (tested at 250, 500, 1000 and 2000 Hz octaves) (see Equation (3)).

(3) $$NRC=\frac{\alpha_{250}+\alpha_{500}+\alpha_{1000}+\alpha_{2000}}{4},$$

Based on the experimental data, the reflection coefficient and the impedance ratio were analyzed in relation to the spectrum of frequencies emitted in the acoustic tube.

#### 2.2.3. Microscopic Anatomy of Wood Samples

For the microscopic analysis, the microscopic preparations were extracted from the samples of the four categories of samples, and then analyzed with the optical Olympus CX 43 microscope (Olympus, Tokyo, Japan), from the Forest Biometrics Laboratory, Faculty of Forestry, “Stefan cel Mare” University of Suceava, Romania. For the present study, this analysis had the role of highlighting the differences in the structure of the samples for the three main sections of the wood.

## 3. Results and Discussions

### 3.1. Morphology and Pore Structure

**Figure 3:**
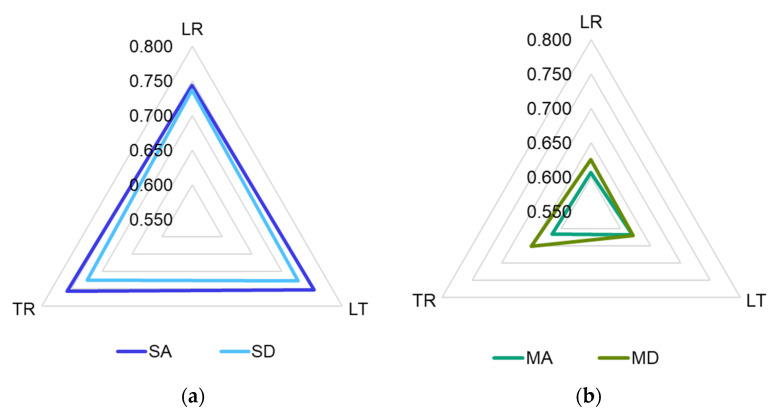
Porosity variation according to the three main sections of the wood: (**a**) spruce wood samples; (**b**) maple wood samples.

As a result of the types of anatomical elements that appear in the three sections of the wood, the morphology of the surfaces of the samples is different and implicitly so too is their porosity. Thus, in the SA samples, the lowest porosity value is found for the LR section. Thus, in the TR section, the porosity is 2% higher compared to the LR section, and in the LT section it is 1.4% higher than for the LR. On the contrary, in class D samples, the highest porosity is recorded in the LR section, and the lowest in the cross section. However, the differences are below 1.6% (Figure 3a). In the case of class A maple wood samples, the highest average porosity value is recorded in the LT section, 1.89% higher than in the LR section, and for class D samples, the highest value is for the TR section, 4.8% higher than in the LT section (Figure 3b).

**Figure 4:**
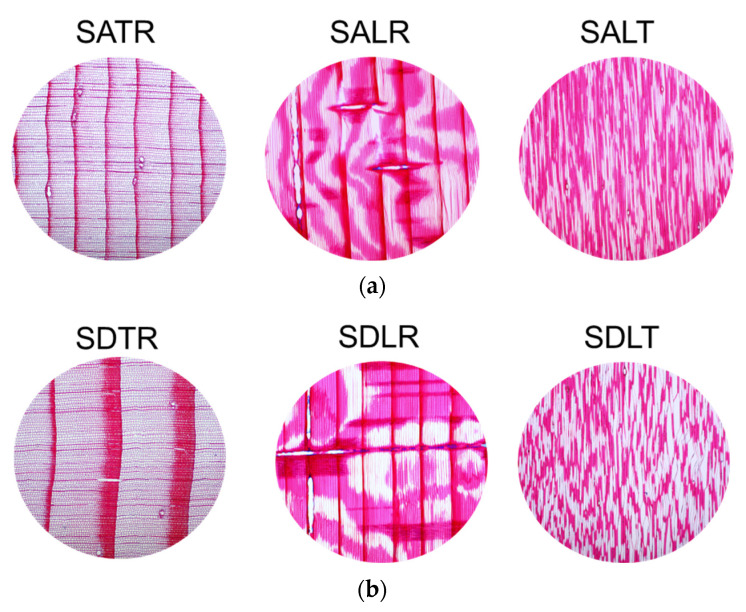
Anatomical features of spruce wood samples (x4 magnification): (**a**) spruce class A, cross section (TR), longitudinal–radial section (LR) and longitudinal–tangential section (LT); (**b**) spruce class D, cross section (TR), longitudinal–radial section (LR) and longitudinal–tangential section (LT).

**Figure 5:**
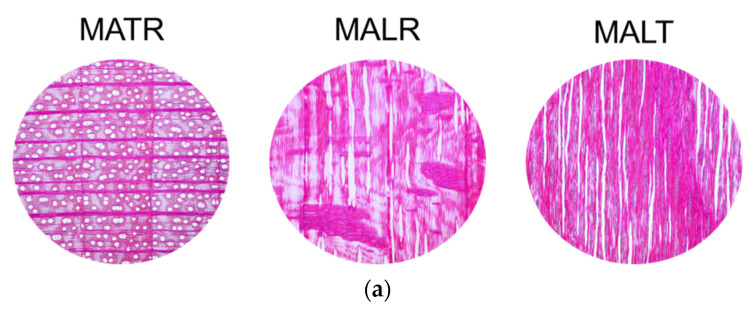

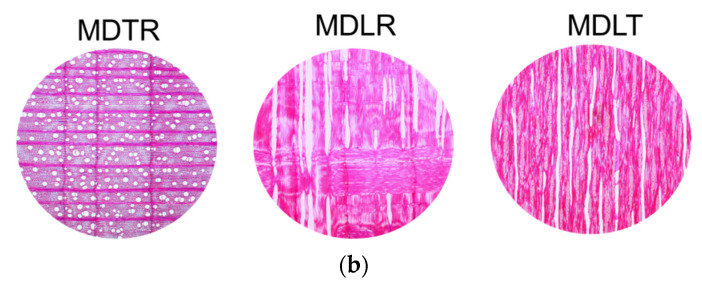
Anatomical features of maple wood samples (x4 magnification): (**a**) maple class A, cross section (TR), longitudinal–radial section (LR) and longitudinal–tangential section (LT); (**b**) maple class D, cross section (TR), longitudinal–radial section (LR) and longitudinal–tangential section (LT).

Table 1 shows the density, porosity and tortuosity values determined according to the methods presented in Section 2.2. As can be seen in Figure 4a,b, in spruce wood, the cross section is characterized by a gradual transition between earlywood and latewood, with resin canals bordered by thick-walled epithelial cells. In the radial–longitudinal section, longitudinal tracheids can be observed, with uniseriate pits and hetero-cellular rays [44,45]. The tangential section is notable for ray formations of 10–15 cells and resin canals with thick-walled epithelial cells. As can be seen in Figure 5a,b, in the maple wood, the cross section is characterized by widely spaced, solitary and multiple radial pores of 2 to 4 (rarely up to 6), depending on the anatomical class. The growth limits of the annual rings are marked by rows of flattened fibers. The fiber walls vary in thickness, forming an irregular pattern [44,45,46]. In the radial section, the maple wood shows distinct spiral thickenings in all the vessels, libriform fibers, homogeneous rays and slightly enlarged pits in the marginal cells of the rays. In the tangential section, single and bisected rays can be seen, with a height varying between 40 and 70 cells.

### 3.2. Sound Absorption Properties

#### 3.2.1. Sound Absorption Coefficient

**Figure 6:**
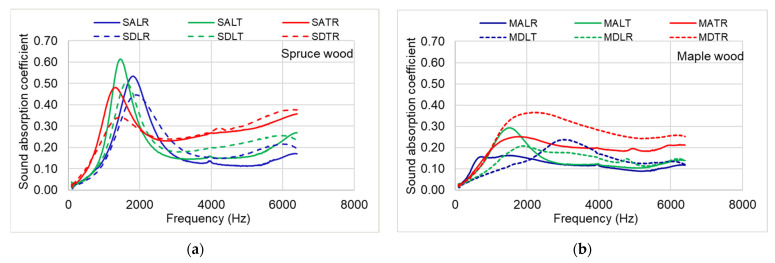
Comparison of average sound absorption coefficient between the three main directions of wood: (**a**) spruce samples; (**b**) maple samples.

The sound absorption coefficient (α) for each frequency between 120 and 6400 Hz was measured using an impedance tube. Figure 6 shows the average graphs of the acoustic absorption coefficient measured for the 10 specimens from each wood species and anatomical quality class. After averaging the values of the sound absorption coefficient, the maximum α value and the frequency at which the maximum α value is recorded were extracted. It can be observed that in the case of spruce samples with the same section, but from different quality classes, the maximum value of the sound absorption coefficient differs but corresponds to the same frequency (Table 2).

In the case of maple wood samples, the values of the absorption coefficient are lower than in spruce wood samples. For the TR and LT sections, the maximum absorption occurs around the same frequency. In the case of the samples analyzed in the LR section, differences are recorded regarding the frequency at which the maximum sound absorption occurs, depending on the quality class. In the radial–longitudinal section, as a result of the wavy fiber specific to class A maple wood, the surface of the samples shows a micro-unevenness as a result of the way the wavy fibers are formed. (Figure 6b).

Between the three main sections, there are differences both in terms of the value of the acoustic absorption coefficient and the frequency at which the maximum value of sound absorption is recorded. Thus, in class A spruce wood, α increases approximately linearly from the value for the LR, LT section to TR, and in samples from class D spruce wood, an inversely proportional behavior is observed (the values decrease from those in the LR, LT section to TR). Maple wood samples show value approximately 1.5 times lower than spruce wood, and the extreme value (minimum or maximum) among the three analyzed sections is obtained for the LT section (maximum in class A samples and minimum in class D samples).

**Figure 7:**
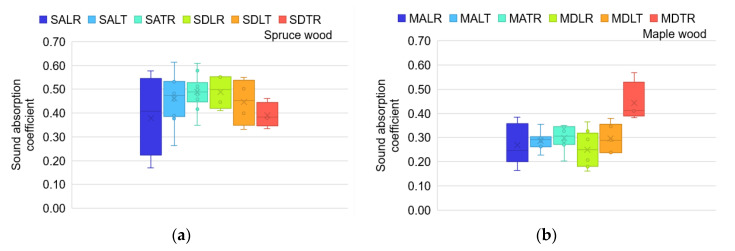
The variation in the maximum sound absorption coefficient: (**a**) spruce samples; (**b**) maple samples.

Figure 7 highlights the variation in the maximum values of the sound damping coefficient. Thus, the dispersion differs depending on the wood species, sample section and the quality class. These values are consistent with those highlighted by [1,2,10], regarding maple wood in the LR section (below 0.25).

**Figure 8:**
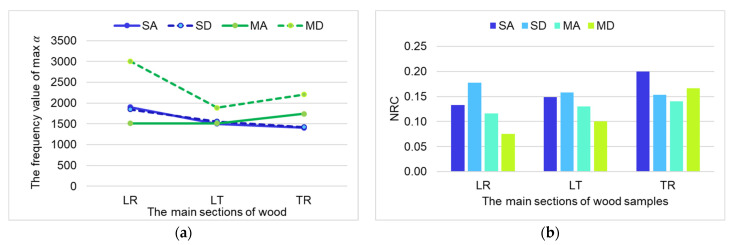
The variation in absorption capability in terms of frequencies for maximum value of sound absorption coefficient (**a**), and of noise reduction coefficient (**b**), respectively.

In the class A maple wood samples, the differences between the three directions related to the frequencies at which the α is maximum, are relatively small (1500–1750 Hz). On the other hand, for the class D samples, the maximum acoustic absorption coefficient varies with section and frequency. The MDLR samples show the best absorption at the frequency of 3000 Hz, the MDLT samples, at the frequency of 1888 Hz, and the MDTR at the frequency of 2200 Hz. Spruce wood samples from both class A and class D show approximately the same frequencies at which the maximum value of the sound absorption coefficient is obtained (Figure 8a). By analyzing the NRC values for the types of samples studied, it can be observed that the highest values are obtained for the samples analyzed on the cross section, regardless of species and quality class. In descending order of the average value of the NRC, the samples with the tangential–longitudinal section follow, and the lowest values are seen for the samples with the radial section (Figure 8b). Only for the spruce wood samples of class D is a higher NRC value found for the longitudinal–radial section.

**Figure 9:**
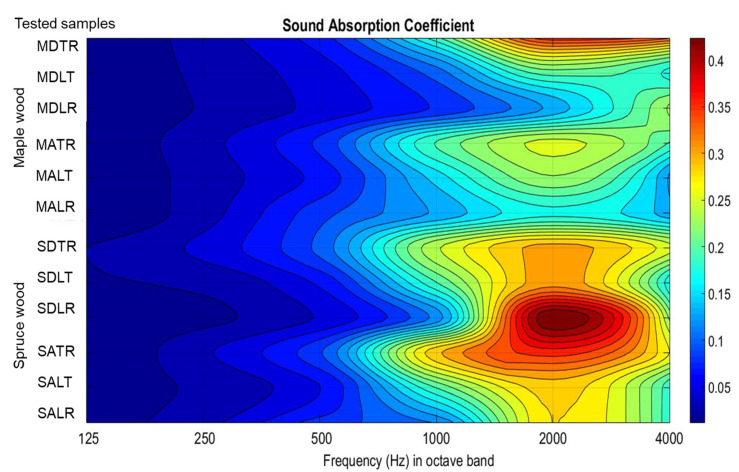
Stratification of the values of the sound absorption coefficient according to the frequencies of octave band, the type of analyzed species, the quality class and the exposed section.

According to Table 3 and Figure 9, it can be seen that the maximum values of the absorption coefficients in octave bands are stratified around the frequency of 2 kHz, regardless of the type of tested specimen. There are differences depending on the species and the analyzed section. This is due to the fact that there are several factors that influence the size of the acoustic absorption coefficient (the different physical and mechanical properties of the three analyzed sections, the chemical composition of the wood, the anatomical differentiation, the size of the pores and the air contained in the cavities of the wood cell or wood material, as was also noticed in [19,20,21,22,27].

Applying the simple Pearson correlation analysis between variables such as density, tortuosity, porosity and the acoustic absorption coefficient for each category of samples, the correlation coefficients in Table 4 and Table 5 resulted. The correlations between density, porosity and tortuosity were excluded, the coefficient being almost ±1, since the porosity and tortuosity values were calculated based on the formulas that include the density in the calculation relationship. 

Thus, it is observed that there are moderate correlations in the case of spruce wood samples; in descending order, it is recorded in the case of SDLR, SDLT and SATR samples. For the maple wood samples, the strongest correlations between the absorption coefficient and the physical properties of the samples are observed in the MDTR, MDLR and MALT samples. These correlations are moderate from the point of view of the obtained values. 

#### 3.2.2. Reflection Coefficient

**Figure 10:**
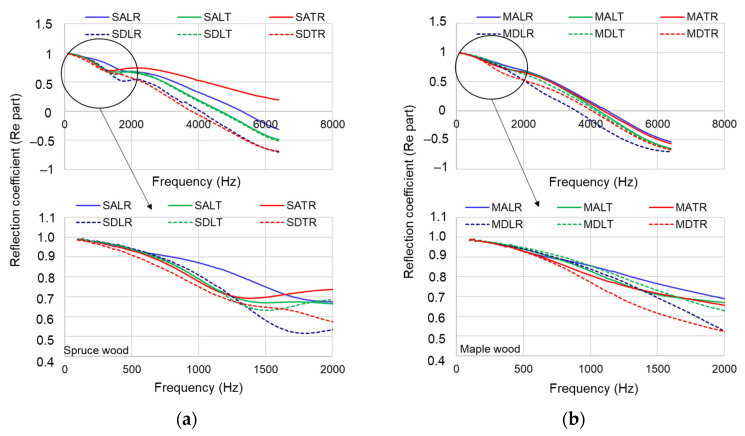
The variation in the reflection coefficient–real part: (**a**) spruce samples; (**b**) maple samples.

The averaged reflection coefficient determined for the tested materials is shown in Figure 10a,b. It can be seen that in the spruce wood samples, the most reflective material is SALR, with a reflection coefficient greater than 0.75 for the range 100–1000 Hz. It can be seen that, in general, all types of samples, regardless of the analyzed sections, are characterized by a high reflection coefficient—over 0.5 for the frequency range between 100 and 2000 Hz.

At high frequencies (above 3800 Hz), all types of spruce wood samples tend to have a negative value of the reflection coefficient, starting with SDLR and SDTR (3.8–4.0 kHz), SALT and SDLT (4500 Hz), respectively, SALR (5500 Hz). In maple wood samples, the reflection coefficient is higher than 0.78–0.8 for the range of 100–1000 Hz, and the differences between quality classes and analyzed sections are smaller than for spruce wood samples. At high frequencies, the maple samples register negative values of the reflection coefficient, starting with the frequency of 3.7 kHz (MDLR), 4 kHz (MDTR), 4.2 kHz (MALT, MDLT) and 4.4–4.5 kHz (MATR and MALR) (Figure 10b).

#### 3.2.3. Acoustic Impedance Ratio

**Figure 11:**
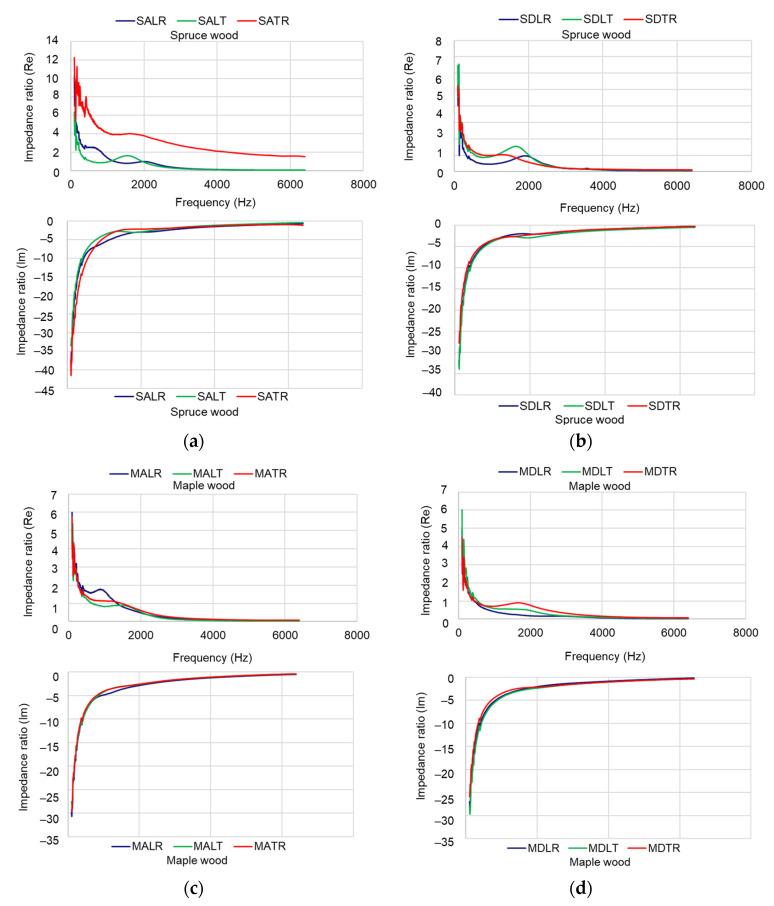
The frequency dependence of the real and imaginary part of the acoustic impedance ratio: (**a**) spruce class A; (**b**) spruce class D; (**c**) maple class A; (**d**) maple class D.

Figure 11 shows the dependence between the real and imaginary parts of the impedance ratio as a function of frequency (in the range of 120 Hz–6.4 kHz).

The values obtained in Figure 11 are the averaged values of the tested samples for each sample category. As noted by [26] in a study on wood samples analyzed for electrical impedance which found that the major differences appear below 2000 Hz, in the present study it appears that the major differences in the acoustic impedance ratio between the three main directions of the wood, but also between the quality classes of the two species, respectively, appear at frequencies below 2 kHz. The two components of the acoustic impedance presented in Figure 11 highlight the behavior of the surface in terms of resistance, through the real component, and of the reactance, through the imaginary component. While the reactance of the samples is similar, regardless of the species and the analyzed section, the resistance of the investigated surfaces differs.

## 4. Conclusions

This study addressed the analysis of the acoustic properties of wood used for musical instruments, from the perspective of absorption, reflection and acoustic impedance. The behaviors of the wood samples in the three main sections of the wood were analyzed. Thus, the following conclusions can be highlighted:

- The absorption coefficient generally does not have a constant value for a certain wood species, varying according to the sound frequency and the angle of incidence of the sound waves on the material. It was concluded that the changes in the sound absorption coefficient indicate a change in the wood pore structure, for which the class of anatomical quality influences the size of this parameter;
- In the longitudinal–radial section, the class A samples, for both spruce and maple wood, presented the lowest values of the sound absorption coefficient, which confirms the use by luthiers of these categories of species and varieties;
- The frequencies at which the maximum values of the sound absorption coefficient were obtained are approximately the same for spruce wood, regardless of the quality class, in contrast to maple wood where there are differences between the frequencies at which the highest acoustic absorption occurs;
- The noise reduction coefficient (NRC) anisotropy ratio between the main sections of the wood are: spruce sample class A, TR:LT:LR: 1.0:1.35:1.50; spruce sample class D, TR:LT:LR: 1:1.16:1.12; maple sample class A, TR:LT:LR: 1:1.18:1.31; maple sample class D, TR:LT:LR: 1:1.66:2.20;
- Due to the anatomical structure, regardless of the analyzed section, both class A and class D maple wood are more homogeneous from the point of view of the reflection coefficient than spruce wood;
- Most variations in the impedance ratio for the analyzed samples are recorded below the frequency of 2000 Hz;
- The closest correlation between porosity and the acoustic absorption coefficient is recorded for the class D spruce wood samples, LR section (Table 4), and for the maple wood samples; also, those from class D, TR, and LR sections show the highest correlation between the sound absorption coefficient and porosity (Table 5).

In conclusion, the study highlights the properties of wood used in the construction of musical instruments from the perspective of reflection and acoustic absorption, being among the few studies that address this issue. Obviously, in future studies, research on the vibration modes, resonance frequency spectrum, damping and quality factor will be addressed, results that will be correlated with those obtained in the study presented in this paper.

## Figures and Tables

**Table 1:** Physical features of samples (average values and standard deviation).

Samples	Wood Species	$\mathrm{Absolutely}\;\mathrm{Dry}\;\mathrm{State}\;\mathrm{Density}\;\rho_{0}$ (kg/m^3^)	Porosity *D_w_*	Tortuosity *k_s_*
SALR	Spruce	384.9 (18)	0.743 (0.012)	1.160 (0.009)
SALT		369.1 (65)	0.754 (0.043)	1.153 (0.030)
SATR		362.4 (32)	0.758 (0.021)	1.149 (0.016)
SDLR		395.5 (18)	0.736 (0.012)	1.165 (0.012)
SDLT		409.6 (9)	0.727 (0.010)	1.173 (0.010)
SDTR		413.2 (11)	0.725 (0.007)	1.175 (0.006)
MALR	Maple	589.3 (10)	0.607 (0.007)	1.283 (0.007)
MALT		572.0 (22)	0.619 (0.015)	1.272 (0.015)
MATR		577.0 (17)	0.615 (0.011)	1.275 (0.012)
MDLR		562.4 (20)	0.625 (0.013)	1.265 (0.013)
MDLT		568.9 (20)	0.621 (0.013)	1.269 (0.013)
MDTR		524.4 (12)	0.650 (0.007)	1.240 (0.007)

**Table 2:** The NRC and maximum values of the sound absorption coefficient for each category of tested samples.

Sample	Wood Species	NRC	Max α	Frequency (Hz) for Max α
SALR	Spruce	0.133	0.288	1904
SALT		0.148	0.357	1504
SATR		0.200	0.456	1408
SDLR		0.178	0.348	1424
SDLT		0.159	0.464	1848
SDTR		0.153	0.435	1560
MALR	Maple	0.116	0.164	1512
MALT		0.130	0.293	1512
MATR		0.141	0.251	1744
MDLR		0.076	0.238	3008
MDLT		0.100	0.209	1888
MDTR		0.166	0.366	2208

**Table 3:** The sound absorption coefficient related to octave band.

Samples	Wood Species	Frequency (Hz)					
		125	250	500	1000	2000	4000
SALR	Spruce	0.022	0.041	0.088	0.117	0.286	0.186
SALT		0.023	0.032	0.063	0.202	0.295	0.188
SATR		0.014	0.043	0.095	0.299	0.363	0.269
SDLR		0.018	0.025	0.048	0.121	0.440	0.230
SDLT		0.028	0.033	0.067	0.199	0.314	0.172
SDTR		0.029	0.051	0.103	0.249	0.309	0.257
MALR	Maple	0.012	0.039	0.091	0.135	0.180	0.139
MALT		0.013	0.037	0.069	0.156	0.232	0.129
MATR		0.016	0.040	0.084	0.202	0.257	0.198
MDLR		0.012	0.031	0.051	0.083	0.138	0.237
MDLT		0.013	0.033	0.055	0.105	0.206	0.177
MDTR		0.013	0.037	0.072	0.193	0.363	0.336

**Table 4:** The correlation coefficients between the sound absorption coefficient and the physical features, in cases of spruce wood samples.

Sound Abs. Coeff.	Density (kg/m^3^)	Porosity (-)	Tortuosity
SATR	−0.387	0.387	−0.390
SALR	−0.055	0.055	−0.063
SALT	0.114	−0.114	0.134
SDTR	0.279	−0.279	0.277
SDLR	0.657	−0.657	0.656
SDLT	−0.512	0.512	−0.513

The colors in the table cells are set based on a 3-color scale, where the minimum value (−1) is blue, the average value (0) is white, and the maximum value (+1) is red. The color tones in the table correspond to values between −1 and +1, highlighting the polarization of values in the mentioned range.

**Table 5:** The correlation coefficients between the sound absorption coefficient and the physical features, in cases of maple wood samples.

Sound Abs. Coeff.	Density (kg/m^3^)	Porosity (-)	Tortuosity
MATR	0.390	−0.390	0.389
MALR	0.136	−0.136	0.137
MALT	−0.487	0.487	−0.487
MDTR	−0.622	0.622	−0.623
MDLR	0.571	−0.571	0.572
MDLT	0.263	−0.263	0.269

The colors in the table cells are set based on a 3-color scale, where the minimum value (−1) is blue, the average value (0) is white, and the maximum value (+1) is red. The color tones in the table correspond to values between −1 and +1, highlighting the polarization of values in the mentioned range.

## Data Availability

Not applicable.
